# Computational Study on Surface Bonding Based on Nanocone Arrays

**DOI:** 10.3390/nano11061369

**Published:** 2021-05-21

**Authors:** Xiaohui Song, Shunli Wu, Rui Zhang

**Affiliations:** 1 School of Mechanical Engineering, Zhengzhou University, Zhengzhou 450001, China; xhsong@foxmail.com; 2 Institute of Applied Physics, Henan Academy of Sciences, Zhengzhou 450008, China; yukephysics@gmail.com

**Keywords:** surface bonding, nanocone arrays, molecular dynamics simulation

## Abstract

Surface bonding is an essential step in device manufacturing and assembly, providing mechanical support, heat transfer, and electrical integration. Molecular dynamics simulations of surface bonding and debonding failure of copper nanocones are conducted to investigate the underlying adhesive mechanism of nanocones and the effects of separation distance, contact length, temperature, and size of the cones. It is found that van der Waals interactions and surface atom diffusion simultaneously contribute to bonding strength, and different adhesive mechanisms play a main role in different regimes. The results reveal that increasing contact length and decreasing separation distance can simultaneously contribute to increasing bonding strength. Furthermore, our simulations indicate that a higher temperature promotes diffusion across the interface so that subsequent cooling results in better adhesion when compared with cold bonding at the same lower temperature. It also reveals that maximum bonding strength was obtained when the cone angle was around 53°. These findings are useful in designing advanced metallic bonding processes at low temperatures and pressure with tenable performance.

## 1. Introduction

Surface bonding is an essential step in device manufacturing and assembly, providing mechanical support, heat transfer, and electrical integration. Traditional surface bonding techniques in electronic assembly strongly rely on high-temperature processes such as reflow soldering, which can lead to undesirable thermal damage, toxic solder materials pollution, and residual stress at the bonding interface [1,2]. Besides, thermo-compression is another widely used bonding approach because it provides intrinsic interconnections and excellent bonding strength. However, high bonding pressure and temperature may result in bond-alignment deviation, high thermal stress, and possible damages to bonded devices [3].

Consequently, to achieve high-density integration, high performance, and low power consumption, new high-level surface bonding schemes have to be developed. Recently, the process compatibility and reliability of cold bonding between surfaces with patterned arrangements of nanowires, nanoparticles, and nanocones have been improved by studies of the effects of lowering the temperature and pressure for bonding [4,5,6,7,8]. Such nanometal bonding methods exhibit high bonding strength and low electrical resistance at the interface at ambient or low temperatures. A maximum adhesion strength of 16.4 N/cm^2^ was achieved using a bent, hook-like nanowire surface fastener, showing a room temperature bonding ability and adaptability to a highly ordered electrode such as the ball grid array (BGA) [9]. In addition, a new copper/polystyrene core/shell nanowire surface fastener showed a higher adhesion strength (~44.42 N/cm^2^) and a much lower contact resistivity (~0.75 × 10^−2^ Ω·cm^2^) [10]. Furthermore, the metallic nano structures exhibit good compatibility with a bendable or stretchable device, and can be fabricated on flexible electronic circuits, making them attractive as adhesive materials for wearable applications [11].

To design a nanometal structure with optimum properties, it is crucial to understand the atomic bonding mechanisms at the nanoscale and accordingly optimize the bonding process. Several mechanisms and theoretical models, such as van der Waals (vdW) interactions [12], surface coalescence [13,14], and Amonton’s first law [15], have been proposed to describe the processes of metal nanowires and nanoparticles-based surface bonding. However, some novel phenomena observed in nanometal-based surface bonding suggest that different nanostructures relate to different bonding mechanisms. All too often, a single mechanism is described as the cause of surface bonding when nanometal bonding may be a combination of mechanisms that contribute to the nanoscale adhesion. It was reported previously that metal nanocones-based surface bonding, which has relative controllability and operational fabrication processes, can be performed at room temperature [16,17]. However, to our knowledge, the discussion of metal nanocones-based surface bonding from the micro mechanism is still rare.

In this work, we explored the atomic interactions that lead to the formation of surface joints between copper nanocones using molecular dynamics (MD) simulations. Conical nanometals with various diameters and heights were then placed in close proximity at different separation distances and contact lengths to form nanoscale surface bonding at the interface. The debonding process and the effect of the temperature on the mechanical properties of the interface bonding between copper nanocones were investigated. 

## 2. Computational Methods

**Figure 1:**
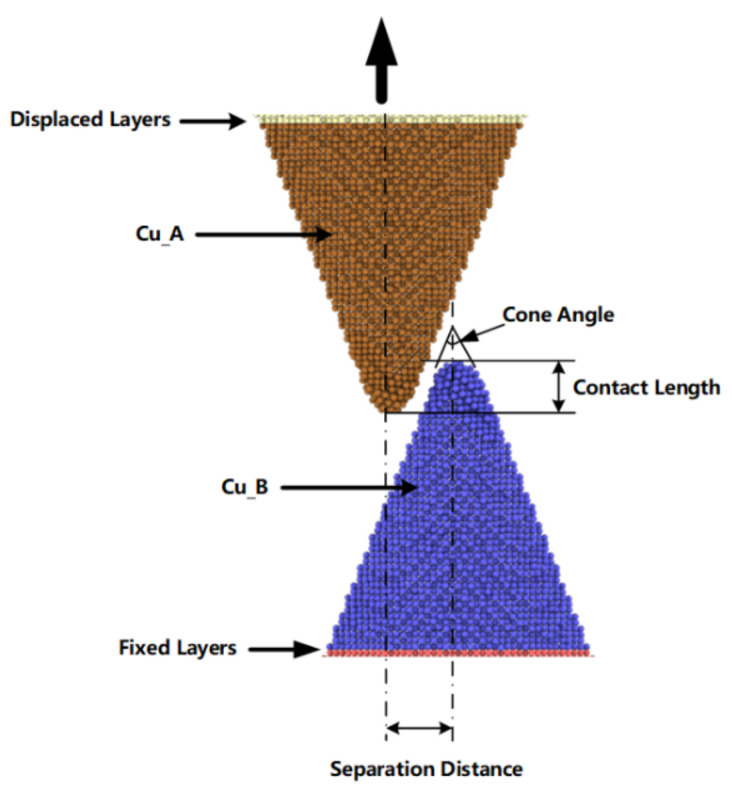
Copper nanocones surface bonding simulation model.

Molecular dynamics (MD) simulation has been proven to be a powerful tool at the nanoscale and is widely used in the studies of nanomaterials [18]. In the present study, MD simulations were used to deal with the bonding and debonding process of the copper nanocones interface joint. Our simulations were based on the massively parallel LAMMPS code [19]. The visualization was based on Open Visualization Tool (OVITO) [20]. In this work, we modeled copper nanocones with various diameters and heights. As shown in Figure 1, the contact length represents the overlap depth of two cones, the separation distance represents the distance between two cone axes. This study involves a combination of embedded atom method (EAM) potential and Lennard-Jones potential [21,22]. The EAM potential [23,24,25] is proven to accurately depict the many-body atomic interactions in metallic systems, and is widely used to simulate the deformation behavior, indentation behavior of thin copper, and Cu composites [26,27,28]. Therefore, the EAM potential was used to calculate the interatomic interactions between copper atoms inside the nanocone; while, in the simulation of the contact process, the Lennard-Jones (LJ) pair potential, which involves nonbond long-range interactions, was used to describe the interactions between copper atoms located at the interface of the adjacent nanocones on the opposite surfaces of the joint. For fcc Cu, an LJ potential with parameters *σ* = 2.228 Å, *ε* = 0.415 eV and a cutoff radius = 4 *σ* was used in the simulation [29,30].

**Figure 2:**
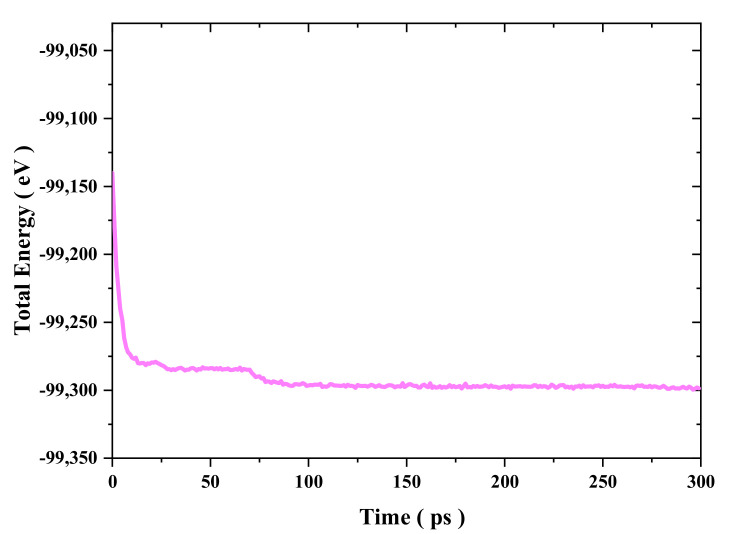
Total energy variation during 300 ps equilibration of a pair of nanocones, as oriented in Figure 1, with an initial axial separation of 20 Å and a contact length of 20 Å.

The initial copper nanocone structure with a diameter of 80 Å and height of 100 Å was cut from perfect bulk face-centered cubic (fcc) crystals with lattice constant of 0.3615 nm by atomsk [31]. A single cone was equilibrated and optimized using the conjugate gradient algorithm to perform an energy minimization of the system for 300 ps, with a time step of 0.5 fs afterwards. Additionally, the two nanocones formed a surface joint with a contact length of 20 Å and a separation distance of 20 Å, as shown in Figure 1, which allowed the two cones to be attracted to each other and form cold bonds under diffusion and intermixing of surface atoms. The configuration contained 29,192 atoms. As shown in Figure 1, the separation distance represents the distance between the parallel axes of the two cones, and the contact length represents overlapping depth of the two cones. During simulation, non-periodic boundary conditions were implemented in each direction. We conducted a surface bonding simulation with the displaced layer and the fixed layer was fixed. As shown in Figure 2, the simulation model underwent an equilibration process for 300 ps in the NVT ensemble at a constant temperature using a Nose–Hoover thermostat with a simulation input temperature of 300 K [32]. 

To analyze the mechanical properties, such as the maximum load that can be sustained by the formed bonding joint, we performed MD simulations for the debonding process under tensile loading. First, the fixed layer was fixed while the displaced layer displaced along the cone axis by 0.1 Å. Second, the strained structure was equilibrated at the constant temperature for 30 ps in the NVT ensemble. The loading force acting on the fixed layer is averaged over the last 5 ps to reduce fluctuations. This process was repeated until the structure fractures.

## 3. Results and Discussion

**Figure 3:**
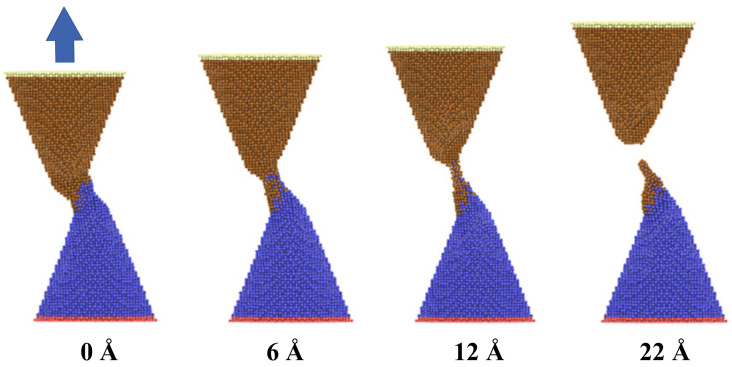
Snapshots of two copper nanocones with an initial axial separation of 20 Å and a contact length of 20 Å at different stages of the uniaxial tensile loading MD simulations.

As shown in Figure 3, the configuration modification of two adjacent copper nanocones during the bonding and the debonding process was investigated. In the initial state, two nanocones formed cold bonds and surface joints at the interface with a certain contact length and separation distance at a temperature of 300 K after equilibrating for 300 ps. Strong adhesion and bonding took place in the contact zone between the two nanocones. As the uniaxial tensile loading was performed, the applied load led to a continuous localized tensile deformation at the contact zone. With increasing tensile deformation, the diameter of the contact zone became thinner until fracturing and separating from one of the nanocones. As noted from the figure, the joint interface was not very clear after surface bonding due to atomic diffusion. It was also found that the adhesion zone with disorderly atoms distribution occurred at the interface of two cones, which was mainly by diffusion and viscous flow bonding. Additionally, one of the nanocones left a number of its atoms adhering to the contacting nanocone after debonding. 

**Figure 4:**
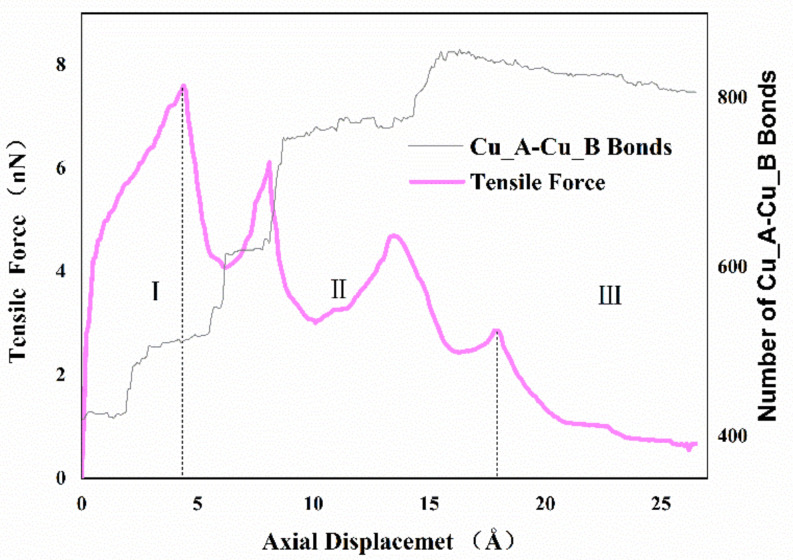
Variation of the tensile loading force and the number of Cu_A-Cu_B bonds with the axial displacement for nanocones, with an initial axial separation of 20 Å and a contact length of 20 Å.

Figure 4 shows the variation of the axial tensile force exerted on the system and the number of Cu_A-Cu_B bonds via the imposed displacement. As illustrated, the curve can be separated into three regimes. In regime one, the force-displacement behaviors present approximate linearity before yielding, and then the value of tensile force increased gradually with increasing imposed displacement until the curve reaches the peak. In regime two, after the first peak, the tensile force decreased rapidly and then raised to another peak with increasing imposed displacement. Subsequently, the tensile force decreased and reached a local minimum at about 10 Å, then the relationship between tensile force and displacement presented a zigzag pattern. Finally, in regime three, the value of tensile force closed to zero until the two cones separated. It appeared that the interface becomes harder and stronger repeatedly during the necking phase in regime two, which is different from the typical material tensile curve. To further study this phenomenon, the number of Cu_A-Cu_B bonds with a cutoff radius of 3.2 Å was used to describe the formation of cold bonds and surface joints at the interface. From the results, increasing the displacement would affect tensile force while increasing Cu_A-Cu_B bonds in the form of steps. During the bonding process, the top atoms of the nanocones were very active, which not only formed the van der Waals force connection but also indicated the formation of atomic diffusion and cold bonds. In the early stages of stretching, both van der Waals interactions and few created bonds induced elastic deformation. With the increase in deformation, the van der Waals force decreased, and the necking behavior made the interface thinner, which resulted in enhanced surface atom diffusion and intermixing of surface atoms between the interfaces. More cold bonds were formed. It is also noted that more created metal bonds accompanied strain hardening, suggesting that the atomic diffusion is helpful to restrain interface failure.

In order to further understand the interface bonding mechanism, the adhesion energy generated by both non-bonded long-range van der Waals force and cold bonds’ interaction at the interface between the two cones during debonding process was investigated. In this study, the adhesion energy could be calculated from Equation (1)

**Figure 5:**
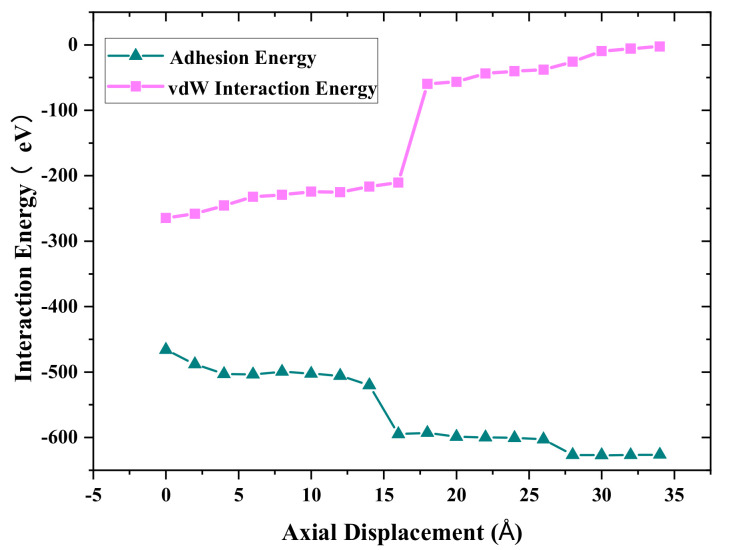
Variation of the adhesion energy and vdW interaction energy with the axial displacement.

E_adhesion_ = E_vdW_ + E_bond_ (1)

where E_adhesion_ is the total interaction energy between Cu_A and Cu_B, E_bond_ is the Cu_A-Cu_B bonds’ interaction energy with a cutoff radius of 3.2 Å, and E_vdW_ is long-range non-bonded van der Waals energy between Cu_A and Cu_B with a cutoff radius of 8.912 Å. Figure 5 shows the adhesion energy and van der Waals energy distributions with different axial displacement during the debonding process. The negative value indicates that the Cu_A and Cu_B atoms attract to each other. The absolute value of the adhesion energy increases with increasing axial displacement. However, the absolute value of van der Waals energy decreases with an increase in axial displacement. The reason is that more cold bonds are formed during the stretching process, as shown in Figure 4, and E_bond_ increases continuously, while the absolute value of long-range van der Waals energy decreases with the increasing distance between Cu_A and Cu_B. It was also found that the contribution of van der Waals energy and metallic bond energy to the adhesion properties is close before the tensile force reaches the maximum. With the further increase in tensile displacement, the bond energy can play a main role in the necking stage. 

**Figure 6:**
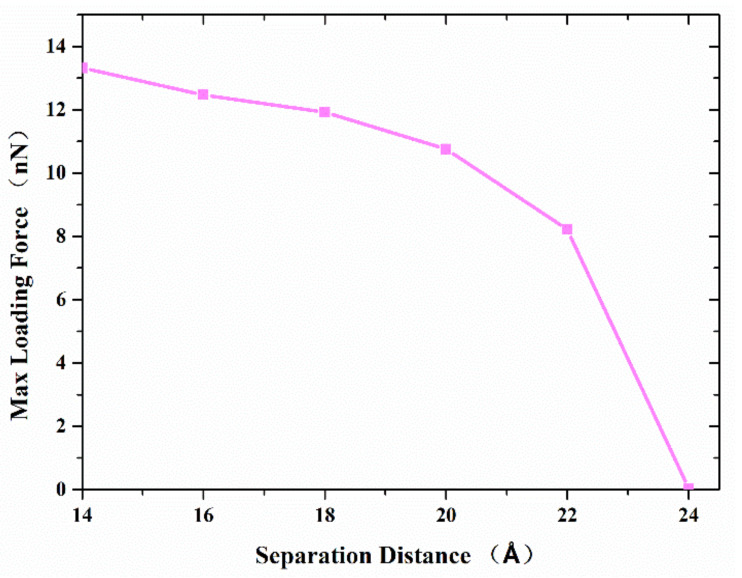
The maximum axial force that the interface can resist with the separation distance.

We also investigated the effect of separation distance and contact length on the joining strength of two copper nanocones with a diameter of 80 Å and a height of 100 Å at a temperature of 300 K. Figure 6 shows the maximum axial tensile loading force that the formed joint between the two nanocones can resist, with the variation of the separation distance. The results show that the maximum axial tensile loading force decreases with the increase in the separation distance. When the separation distance is more than 20 Å, the maximum axial tensile loading force decreases more greatly. This difference should be ascribed to the following reasons: (1) Both van der Waals force and atomic diffusion affect the maximum axial tensile loading force, and the closer the distance is, the greater the influence of atomic diffusion; (2) beyond a certain separation, diffusion ceases and the van der Waals attraction, which at this point plays the dominant role in joint formation, decreases, leading to a steeper decrease in maximum tensile force. 

**Figure 7:**
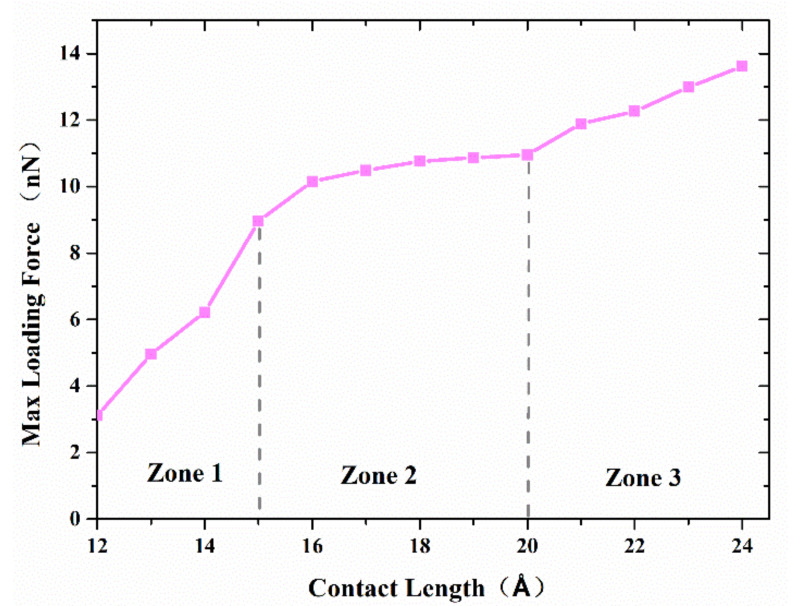
The maximum axial force that the interface can resist with separation distance of 20 Å and various contact length.

**Figure 8:**
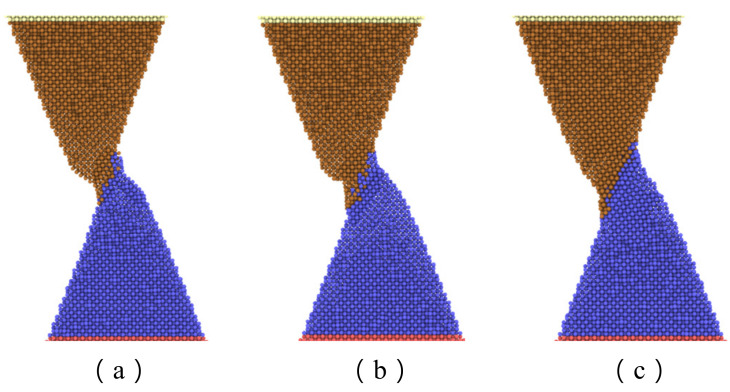
Snapshots of two copper cones forming a bonding interface with a separation distance of 20 Å and various contact lengths: (**a**) 15 Å; (**b**) 20 Å; (**c**) 25 Å.

Figure 7 shows the maximum axial force that the interface can resist with separation distance of 20 Å and various contact length. The curve can be divided into three zones. The first zone, with a contact length less than 15 Å, is concerned with a small interface region and limited adherence of active atoms at the tip of nanocones, as shown in Figure 8a. The slope of the curve in this region is the highest of the three regions. The reason may be ascribed to relatively large numbers of disordered active atom at the cone tips, which diffuse in increasing numbers to form cold bonds as the contact length increases. In the second zone, the increase in maximum axial tensile loading force is due to more atoms on the surface of the cones being involved in forming the joint as shown in Figure 8b, and the increment of the number of metal bonds formed by atomic diffusion is smaller than that of the first zone. Additionally, the increased effective contact area increases the van der Waals force. In the third zone, where the contact length is more than 20 Å, larger contact areas are involved in forming the adherence joint, as shown in Figure 8c. On the other hand, to achieve a larger contact length, larger preloading forces, which will contribute to the diffusion of interface atoms, are typically used. As a result, more cold bonds are formed at the interface so that the slope of the curve in this region is higher than that of the second zone.

**Figure 9:**
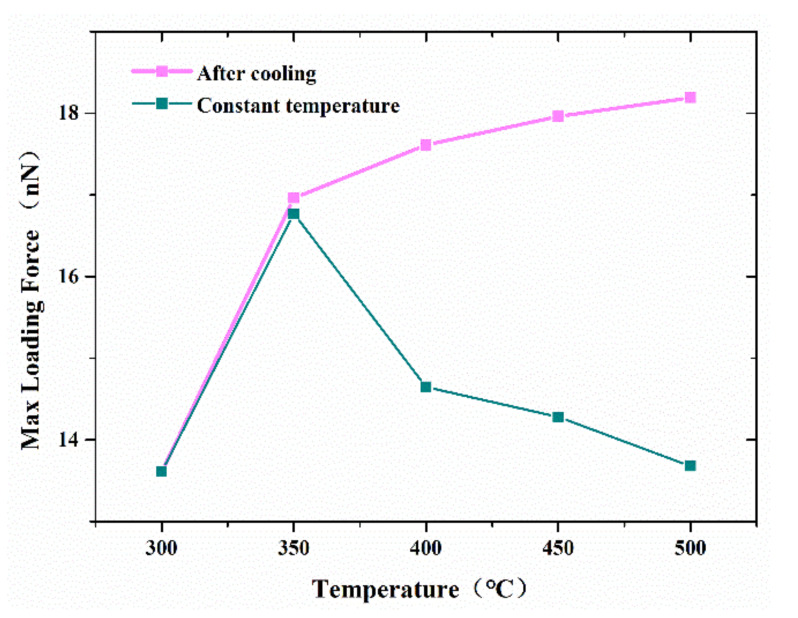
Effect of temperature on the strength of the formed joints between two cones.

The effect of temperatures ranging from 300 to 500 K on the strength of the formed joints between two cones was also investigated in this study. Figure 9 shows the variation of the maximum tensile force that the contact nanocones can sustain with increased temperature. For simulation at a constant temperature, the maximum tensile force increases with the increase in temperature as the temperature is lower than 350 °C. Additionally, the maximum tensile force keeps on decreasing with the increase in temperature. It appeared that the increase in temperature could significantly promote the diffusion of interface atoms, resulting in increasing joint strength. However, when the temperature was increased, the maximum tensile forces were continuously decreased. This suggested that higher temperature promotes melting of the joint, and therefore weak resistance to a tensile force. On the other hand, when the adhesive joint was cooled to 300 °C before debonding, the maximum tensile force kept on increasing with the increase in temperature, as shown in Figure 7. This result illustrates the significant effect of temperature on the performance of surface bonding based on metal nanocones, and will thus help to tune the joint strength for high application.

**Figure 10:**
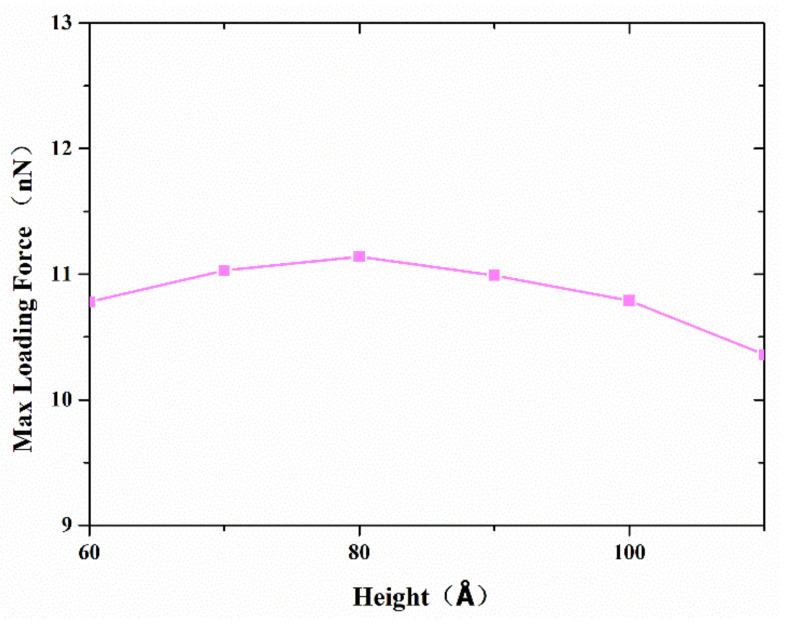
Effect of height of the cone on the strength of the formed joints between two cones with diameter 80 Å, separation distance 20 Å, and contact length 20 Å.

As mentioned above, both atom diffusion and Van der Waals force depending on contact configuration determine the joint strength. Therefore, various copper nanocones, coupled with different diameters and heights, were investigated to find out the effect of surface structure on bonding strength. Serial simulations were performed with a diameter of 80 Å, a separation distance of 20 Å, and a contact length of 20 Å. Figure 10 shows the variation in the maximum tensile force with different heights. The maximum tensile strength increases when the height is lower than 80 Å. However, the maximum tensile strength reaches its highest value at a cone height of about 80 Å, which corresponds to a cone angle of 53°, and decreases thereafter. From a geometric point of view, a larger effective contact area and shorter vertical distance between two surfaces were obtained, with an increasing cone angle corresponding to a lower height diameter ratio. This helps to improve the interface bonding strength. On the other hand, a larger cone angle means that there are relatively few disordered active atoms on the tip, which will reduce atoms’ diffusion during the formation of the joint.

## 4. Conclusions

In this study, we developed a molecular dynamics analysis model of surface bonding based on metal nanocones arrays. The influences of separation distance, contact length, temperature, and size of the cones were examined. Specifically, the focus of this study was devoted to examining and characterizing the resistance capacity to tension force and failure mechanisms associated with the above parameters. The results reveal that both van der Waals interactions and surface atoms’ diffusion are essential constituents that determine the bonding strength. The debonding process of different failure mechanisms is related to their deformation behavior in different regimes. Noteworthy is the fact that more created metal bonds accompanied strain hardening during debonding, which is helpful to restrain interface failure. It is also shown that increasing contact length and decreasing separation distance can simultaneously contribute to increasing the bonding strength. Additionally, increasing the operating temperature and subsequent cooling would contribute to atoms’ diffusion and bonding strength increasing. It also reveals that the maximum bonding strength was obtained when the cone angle was around 53°. Decidedly, the work offers bonding and debonding mechanisms involved at the atomic level for surface bonding based on metal nanocone arrays, which is helpful to design and optimize the surface bonding process.

## Data Availability

Data available in a publicly accessible repository.

## References

[1] Shin H., Han E., Park N., Kim D. (2018). Thermal residual stress analysis of soldering and lamination processes for fabrication of crystalline silicon photovoltaic modules. Energies.

[2] Vianco P.T. (2019). A review of interface microstructures in electronic packaging applications: Soldering technology. JOM.

[3] Ko C.T., Chen K.N. (2012). Low temperature bonding technology for 3d integration. Microelectron. Reliab..

[4] Ju Y., Amano M., Chen M. (2012). Mechanical and electrical cold bonding based on metallic nanowire surface fasteners. Nanotechnology.

[5] Wang P., Ju Y., Chen M., Hosoi A., Song Y., Iwasaki Y. (2013). Room-temperature bonding technique based on copper nanowire surface fastener. Appl. Phys. Express.

[6] Li J., Yu X., Shi T., Cheng C., Fan J., Cheng S., Liao G., Tang Z. (2017). Low-temperature and low-pressure cu–cu bonding by highly sinterable cu nanoparticle paste. Nanoscale Res. Lett..

[7] Wang H., Leong W.S., Hu F., Ju L., Su C., Guo Y., Li J., Li M., Hu A., Kong J. (2018). Low-temperature copper bonding strategy with graphene interlayer. ACS Nano.

[8] Lee J., Lee I., Kim T.S., Lee J.Y. (2013). Efficient welding of silver nanowire networks without post-processing. Small.

[9] Toku Y., Ichioka K., Morita Y., Ju Y. (2019). A 64-pin nanowire surface fastener like a ball grid array applied for room-temperature electrical bonding. Sci. Rep..

[10] Wang P., Yang J. (2015). Room-temperature electrical bonding technique based on copper/polystyrene core/shell nanowire surface fastener. Appl. Surf. Sci..

[11] Yuhki T., Keita U., Yasuyuki M., Yang J. (2018). Nanowire surface fastener fabrication on flexible substrate. Nanotechnology.

[12] Wang P., Ju Y., Cui Y., Hosoi A. (2013). Copper/parylene core/shell nanowire surface fastener used for room-temperature electrical bonding. Langmuir.

[13] Kang Z., Wu B. (2018). Coalescence of gold nanoparticles around the end of a carbon nanotube: A molecular-dynamics study. J. Manuf. Process..

[14] Cha S.H., Park Y., Han J.W., Kim K., Kim H.S., Jang H.L., Cho S. (2016). Cold welding of gold nanoparticles on mica substrate: Self-adjustment and enhanced diffusion. Sci. Rep..

[15] Mo Y., Turner K.T., Szlufarska I. (2009). Friction laws at the nanoscale. Nature.

[16] Xu P., Hu F., Shang J., Hu A., Li M. (2016). An ambient temperature ultrasonic bonding technology based on cu micro-cone arrays for 3d packaging. Mater. Lett..

[17] Wang H., Ju L., Guo Y., Mo X., Ma S., Hu A., Li M. (2015). Interfacial morphology evolution of a novel room-temperature ultrasonic bonding method based on nanocone arrays. Appl. Surf. Sci..

[18] Li Q., Fu T., Peng T., Peng X., Liu C., Shi X. (2016). Coalescence of cu contacted nanoparticles with different heating rates: A molecular dynamics study. Int. J. Mod. Phys. B Condens. Matter Phys. Stat. Phys. Appl. Phys..

[19] Plimpton S. (1995). Fast parallel algorithms for short-range molecular dynamics. J. Comput. Phys..

[20] Stukowski A. (2010). Visualization and analysis of atomistic simulation data with OVITO–the Open Visualization Tool. Model. Simul. Mater. Sci. Eng..

[21] Ren X., Li X., Huang C., Yin H., Wei F. (2020). Molecular dynamics simulation of thermal welding morphology of ag/au/cu nanoparticles distributed on si substrates. Ferroelectrics.

[22] Heinz H., Vaia R.A., Farmer B.L., Naik R.R. (2008). Accurate simulation of surfaces and interfaces of face-centered cubic metals using 12-6 and 9-6 lennard-jones potentials. J. Phys. Chem. C.

[23] Foiles S.M., Baskes M.I., Daw M.S. (1986). Embedded-atom-method functions for the fcc metals Cu, Ag, Au, Ni, Pd, Pt, and their alloys. Phys. Rev. B.

[24] Mishin Y., Mehl M., Papaconstantopoulos D., Voter A., Kress J. (2001). Structural stability and lattice defects in copper: Ab initio, tight- binding, and embedded-atom calculations. Phys. Rev. B.

[25] Hao H., Lau D. (2017). Atomistic modeling of metallic thin films by modified embedded atom method. Appl. Surf. Sci..

[26] Weng S., Ning H., Fu T., Hu N., Zhao Y., Huang C., Peng X. (2018). Molecular dynamics study of strengthening mechanism of nanolaminated graphene/cu composites under compression. Sci. Rep..

[27] Hansson P. (2016). Influence of surface roughening on indentation behavior of thin copper coatings using a molecular dynamics approach. Comput. Mater. Sci..

[28] Zhou H., Li J., Xian Y., Hu G., Li X., Xia R. (2018). Nanoscale assembly of copper bearing-sleeve via cold-welding: A molecular dynamics study. Nanomaterials.

[29] Liu T., Liu G., Wriggers P., Zhu S. (2009). Study on contact characteristic of nanoscale asperities by using molecular dynamics simulations. J. Tribol..

[30] Jung S.C., Suh D., Yoon W.S. (2010). Molecular dynamics simulation on the energy exchanges and adhesion probability of a nano-sized particle colliding with a weakly attractive static surface. J. Aerosol Sci..

[31] Pierre H. (2015). A tool for manipulating and converting atomic data files. Comput. Phys. Comm..

[32] Hickman J., Mishin Y. (2016). Temperature fluctuations in canonical systems: Insights from molecular dynamics simulations. Phys. Rev. B.

